# *Bordetella pertussis* Isolates, Finland

**DOI:** 10.3201/eid1101.040632

**Published:** 2005-01

**Authors:** Johanna Mäkinen, Jussi Mertsola, Frits R. Mooi, Shirley Van Amersfoorth, Heikki Arvilommi, Matti K. Viljanen, Qiushui He

**Affiliations:** * National Public Health Institute, Turku, Finland;; † Turku Graduate School of Biomedical Sciences, Turku, Finland;; ‡ University of Turku, Turku, Finland;; § National Institute of Public Health and the Environment, Bilthoven, the Netherlands

**Keywords:** letter, Bordetella pertussis, whooping cough, epidemiology, molecular typing, pertactin, pertussis toxin

**To the Editor:** Pertussis, or whooping cough, is a highly contagious respiratory disease in humans caused by *Bordetella pertussis*. Reemergence of pertussis has been observed in many countries with high vaccination coverage. In the United States, reported cases of pertussis in adolescents and adults have increased since the 1980s, despite increasingly high rates of vaccination in infants and children (*1*). At the same time, clinical *B. pertussis* isolates have become antigenically divergent from vaccine strains (*2**,**3*). This observation has raised the question of whether vaccination has caused selection for the variant strains, and whether the reemergence of pertussis in vaccinated populations is due to vaccination not protecting against these antigenic variants as effectively as it protects against vaccine type strains. On the other hand, vaccine-induced immunity wanes over time, and pertussis is not only a childhood disease but also a frequent cause of prolonged illness in adults and adolescents today (*4*).

In Finland, children are vaccinated with diphtheria-tetanus whole-cell pertussis vaccine at 3, 4, and 5 months, and at 20 to 24 months of age. The whole-cell vaccine contains 2 strains and has remained unchanged since 1976. The vaccine strain 18530 contains fimbriae 3 (Fim3), pertussis toxin S1 subunit D (PtxS1D), and pertactin 1(Prn1); the other vaccine strain, 1772, contains Fim2,3, PtxS1B, and Prn1. Ninety-six percent of Finland's population has been vaccinated with 4 doses of pertussis vaccine. The incidence of pertussis is highest in infants <1 year of age and in schoolchildren from 6 to 14 years old, although about 30% of the cases occur in adults older than 20 years. In Finland, as in many other countries with large-scale vaccination programs, several outbreaks of pertussis occurred in the 1990s. We studied prospectively 3 pertussis outbreaks in 2 elementary schools and 1 municipality in southwestern Finland (*5**,**6*). The aim of the study was to characterize the strains circulating and causing outbreaks and to track the transmission of *B. pertussis* during these outbreaks.

Sample were collected and primary cultures were done as described earlier (*5**,**6*). The outbreaks took place in 3 rural municipalities: in 1992, in Paimio (Table 1) with 9,900 inhabitants; in 1995, in Oripää (Table 2) with 1,400 inhabitants; and in 1996, in Rusko (Table 3) with 3,500 inhabitants. The isolates were obtained from schools and local health centers. In addition, 1 isolate was obtained from a household contact (Table 3). Most of the cases occurred in schoolchildren >8 years of age and in adults.

**Table 1:** Characteristics of the patients and *B. pertussis* isolates of the Paimio outbreak*

Isolate PRCB	School	Age of patient (y)	Sampling time (m/d/y)	Days of cough at the sampling	Fim	*ptxS1*	*prn*	*tcfA*	RFLP	PFGE
9	†	4	09/05/92	Asymptomatic	2	A	4	2	29	1
10	A	8	09/17/92	14	2	A	2	2	12	2
12	A	8	09/23/92	8	2	A	2	2	29	2
13	A	8	09/23/92	12	2	A	2	2	29	2
14	†	5	09/29/92	7	2	A	2	2	Nd	2
15	†	36	09/29/92	1	2	A	2	2	29	2
18	†	42	10/20/92	10	2	A	2	2	Nd	2
17	A	10	10/20/92	7	2	A	2	2	29	2
20	A	11	10/21/92	7	2	A	2	2	29	2
19	†	50	10/27/92	1	2	A	2	2	Nd	2
21	A	9	10/29/92	5	2	A	2	2	29	2
22	A	10	11/03/92	5	2	A	2	2	29	2
24	A	10	11/03/92	7	2	A	2	2	29	2
25	A	11	11/03/92	14	2	A	2	2	29	2
23	A	9	11/03/92	15	2	A	2	2	29	2
27	A	12	11/03/92	15	2	A	2	2	29	2
26	A	12	11/03/92	16	2	A	2	2	29	2
28	A	12	11/03/92	21	2	A	2	2	29	2
29	†	29	11/09/92	7	2	A	2	2	Nd	2
31	A	12	11/13/92	3	2	A	2	2	Nd	2
30	A	8	11/18/92	8	2	A	2	2	Nd	2
35	†	36	11/20/92	7	2	A	2	2	29	2
34	B	11	11/20/92	7	2	A	3	3	Nd	3
38	B	11	11/24/92	3	2	A	3	3	29	3
40	B	12	11/24/92	2	2,3	A	3	3	Nd	3
39	B	11	11/24/92	5	2,3	A	3	3	29	3

*m, month; d, day, y, year; Fim, serotype; *ptxS1*, pertussis toxin subunit 1 allele; *prn*, pertactin allele; *tcfA*, tracheal colonization factor allele; RFLP, restriction fragment length polymorphism type; PFGE, pulsed-field gel electrophoresis pattern; Nd, not determined †does not attend school

**Table 2:** Characterization of the patients and *B. pertussis* isolates of the Oripää school outbreak*

Isolate PRCB	School	Age of patient (y)	Sampling time (m/d/y)	Days of cough at the sampling	Fim	*ptxS1*	*prn*	*tcfA*	RFLP	PFGE
170	C	9	11/17/95	7	2	A	3	2	29	4
171	C	8	11/17/95	14	2	A	2	2	29	4
172	C	9	11/17/95	Asymptomatic	2	A	2	2	Nd	4
173	C	10	11/17/95	7	2	A	2	2	Nd	4

*m, month; d, day, y, year; Fim, serotype; *ptxS1*, pertussis toxin subunit 1 allele; *prn*, pertactin allele; *tcfA*, tracheal colonization factor allele; RFLP, restriction fragment length polymorphism type; PFGE, pulsed-field gel electrophoresis pattern; Nd, not determined

**Table 3:** Characterization of the patients and *B. pertussis* isolates of the Rusko school outbreak*

Isolate PRCB	School	Age of patient (y)	Sampling time (m/d/y)	Days of cough at sampling	Fim	*ptxS1*	*Prn*	*tcfA*	RFLP	PFGE
191	D	14	09/26/96	11	3	A	2	2	Nd	5
193	D	13	10/11/96	8	2	A	2	2	Nd	6
194	D	13	10/11/96	?	2	A	2	2	Nd	6
192	D	13	10/11/96	0	2	A	2	2	29	6
197	D	13	10/11/96	4	2	A	2	2	Nd	6
209	D	13	10/11/96	2	2	A	2	2	29	6
195	D	13	10/11/96	8	2	A	2	2	29	6
196†	D	13	10/11/96	3	2	A	3	2	29	6
198	D	17	10/17/96	Asymptomatic	2	A	2	2	Nd	6
215	D	13	10/19/96	2	2	A	2	2	29	6
203	D	13	10/21/96	Asymptomatic	2	A	2	2	29	6
202	D	12	10/21/96	5	2	A	2	2	29	6
206	D	16	11/06/96	14	2	A	2	2	Nd	6
208	D	15	11/12/96	5	2	A	2	2	Nd	6
212	D	14	11/14/96	7	2	A	2	2	Nd	6
219†	‡	4	11/22/96	3	2	A	2	2	Nd	7

*m, month; d, day, y, year; Fim, serotype; *ptxS1*, pertussis toxin subunit 1 allele; *prn*, pertactin allele; *tcfA*, tracheal colonization factor allele; RFLP, restriction fragment length polymorphism type; PFGE, pulsed-field gel electrophoresis pattern; Nd, not determined. †family member. ‡does not attend school.

Various DNA fingerprinting techniques, such as IS*1002*-based restriction fragment length polymorphism (IS*1002*-RFLP) and pulsed-field gel electrophoresis (PFGE) have been used to study *B. pertussis* isolates (*7**–**10*). DNA polymorphism analysis of *prn* and *ptxS1* has previously been used as a typing method for detecting antigenic shifts (*2**,**3**,**8*). In addition to *prn* and *ptxS1*, only tracheal colonization factor (*tcfA*), a surface–associated protein involved in the adhesion of *B. pertussis* to host cells, has been found to be polymorphic in recent *B. pertussis* isolates (*3*). The isolates were typed as described earlier (*8**,**10*).

Of the 46 isolates, 43 (94%) expressed Fim2, 2 (4%) expressed both Fim2 and Fim3, and 1 (2%) expressed Fim3 (Tables 1, 2 and 3). The predominant *prn* allele in all 3 outbreaks was *prn2*, contained by 39 (85%) of the isolates. Six (13%) isolates contained *prn3* and 1 (2%) isolate contained *prn4*. All isolates contained the *ptxS1A* allele. The predominant *tcfA* allele was *tcfA2*, contained by 42 (91%) of the isolates. Four (9%) isolates contained *tcfA3*. The *tcfA3* allele was observed only in isolates with *prn3*. All but 1 of the 27 isolates subjected to the *IS1002*-RFLP analysis had the same pattern.

Seven PFGE patterns were found among the 46 isolates studied. The isolates were considered to be closely related, as the differences between the patterns were small, differing by 1 or 2 bands. Three PFGE patterns were found in both Paimio and Rusko. A major pattern was circulating in each of the schools A, B, and C, which indicates that pertussis is effectively transmitted in schools. However, in school D, the isolate from the index patient had PFGE pattern 5, whereas the rest of the isolates from patients in school D had pattern 6. In addition, the 1 isolate obtained from a household contact had a distinct PFGE pattern, 7. Similarly, in Paimio, the isolate from the index patient had a distinct PFGE pattern, 1. These findings, as well as the fact that 3 PFGE patterns were found in both Paimio and Rusko, indicate that several *B. pertussis* strains may have been circulating simultaneously in these small communities.

Our results suggest that *ptxS1* is not a useful marker in outbreaks to detect antigenic shifts. IS*1002*-RFLP was less discriminative than *Xba*I PFGE, which agree with results of previous studies (*8*). Most cases occurred in schoolchildren and adults, confirming epidemiologic findings from other countries with vaccination programs. Our results support the earlier observation that the recent *B. pertussis* isolates are antigenically different from vaccine strains. Several *B. pertussis* strains could circulate simultaneously even in small communities, and only some strains, possibly with increased fitness, are capable of spreading effectively.
